# The mechanism and consequences of chromatin assembly and disassembly

**DOI:** 10.1186/1756-8935-6-S1-O1

**Published:** 2013-03-18

**Authors:** Jessica Tyler

**Affiliations:** 1 UTMD Anderson Cancer Center, Houston, TX, USA

## 

The Eukaryotic genome is packaged together with histone proteins to form the nucleoprotein structure called chromatin. This packaging of DNA into chromatin is critical to prevent inappropriate access to the DNA in order to enable the fundamental processes of the genome, such as gene expression, DNA repair and DNA replication, to be highly regulated. While many groups study histone modifications on the N-terminal histone tails and ATP-dependent chromatin remodeling, we study the ultimate end point of all of these chromatin dynamics, which is the physical removal of histones from the DNA, or their establishment or assembly onto the DNA. Using a combination of biochemistry, structural biology, molecular genetics in budding yeast and tissue culture studies we have identified and characterized the histone chaperones that mediate chromatin assembly and disassembly during replication, repair and gene expression, as well as revealing that these processes are regulated by histone modifications that influence histone-DNA contacts. We have also uncovered that the process of mitotic aging is accompanied by a global loss of approximately 70% of the histones from the genome, presumably leading to increased inappropriate gene expression and genomic instability. At this meeting, we will present our latest findings on chromatin assembly and disassembly.

